# Strengthening recovery, enduring sleep. An ecologically valid assessment of sleep quantity and quality in hybrid athletes: does training mode matter?

**DOI:** 10.1007/s00421-026-06187-9

**Published:** 2026-02-28

**Authors:** Alex Buoite Stella, Fabio D’Andrea, Manuela Deodato, Luigi Murena, Raffaele Sabot, Miriam Martini, Shawnda A. Morrison, Miloš Ajčević

**Affiliations:** 1 Department of Medicine, Surgery and Health Sciences, University of Trieste, Strada di Fiume 447, Trieste, 34149 Italy; 2 Faculty of Sport, University of Ljubljana, Gortanova ulica 22, Ljubljana, 1000 Slovenia; 3 Department of Engineering and Architecture, University of Trieste, via Alfonso Valerio 10, Trieste, 34100 Italy

**Keywords:** sleep, high performance sport, exercise, cardiac function, recovery

## Abstract

**Purpose:**

This study assessed sleep characteristics in a sample of experienced “hybrid” athletes (i.e., athletes who participate and train for competitions that combine endurance and resistance tasks, e.g., Hyrox, Cross-Fit) to investigate the influence of training mode (i.e., resistance training and endurance training) on sleep quality, quantity, and architecture.

**Methods:**

Eight male “Hyrox” athletes (23–32 years) wore a validated, non-invasive, ambulatory sleep monitoring device after completing either a primarily resistance or endurance training bout, performed within a two-week period, consisting of a total two resistance and two endurance post-training nights evaluated. Objectively-measured sleep parameters analysed included: total sleep time (TST), sleep onset latency (SOL), wake after sleep onset (WASO), sleep architecture (sleep stages proportion), and the number and duration of cardiac arousals occurring before and after sleep onset.

**Results:**

Despite reporting ‘good’ overall sleep quality, hybrid athletes experienced shorter TST (~ 6.6 h) and spent ~ 5% less time in REM compared to reference non-athletes, but in-line with other sport populations. Compared to endurance training, resistance training was associated with longer SOL (29 vs. 10 min, *p* = 0.003) and shorter WASO (31 vs. 48 min, *p* = 0.008), with corresponding differences in cardiac arousals before and after sleep onset (13 vs. 5 events before SO, 81 vs. 99 events after SO, respectively, *p* = 0.006 and *p* = 0.010).

**Conclusion:**

Hybrid athletes presented sleep characteristics that differed depending on the training mode of their exercise bout. In particular, sleep latency and WASO time were positively associated with cardiac autonomic responses before and after sleep onset.

**Supplementary Information:**

The online version contains supplementary material available at 10.1007/s00421-026-06187-9.

## Introduction

Sleep as a physiological process has been recognized as particular importance for athletes, considering its critical influence on one’s performance and recovery. Alterations to normal sleep function can be found in athletes, in particular élite athletes, since they are often characterized by reduced sleep duration and poor sleep quality (Walsh et al. 2021). Since poor sleep is commonly experienced after intense training, as well as before competition (Gupta et al. 2017), it has been suggested that sleep quality might depend on some non-sports-specific factor(s), such as societal factors, in addition to sports-specific factors like high training loads, short- and long-haul travels (Zubac et al. 2020), evening competitions (after 18:00) and early morning trainings (before 08:00) (Walsh et al. 2021). In general, acute exercise has been suggested to marginally affect sleep architecture, reporting an increase in slow wave sleep and a delay of the onset of rapid eye movement (REM) sleep (Korkutata et al. 2025). Moreover, although the impact of the training time is still debated, with some studies reporting only a minimal effect of evening exercise on sleep (Stutz et al. 2019), exercise bouts ending more than 4 h before sleep onset seem not to be associated with changes in sleep, regardless of strain (Leota et al. 2025).

Regarding the effects of specific exercise modalities, whereas the effects of chronic resistance exercise have been suggested to improve all aspects of sleep, and in particular sleep quality, the acute effects of resistance exercise remain understudied, yielding inconsistent findings (Kovacevic et al. 2018; Korkutata et al. 2025). Endurance training, in contrast to resistance training, has been found to increase sleep electroencephalography activity associated with hyperarousal during sleep, and produce higher cortisol levels, hypothesizing a hyperarousal effect of endurance exercise when performed in the evening (Perrier et al. 2024). High-intensity exercise (HIE) is generally discouraged before bedtime, since it has been reported to decrease REM sleep compared to no exercise, especially when acutely performed ~ 0.5–4 h before sleep onset. Regular evening HIE does not appear to disrupt nighttime sleep (Frimpong et al. 2021). The inconsistency of these study results has often been suggested to be due to the heterogeneity of the training protocols, and the observed populations (Stutz et al. 2019; Thomas et al. 2020; Yue et al. 2022; Korkutata et al. 2025).Specifically, directly comparing the effects of different training modalities (i.e., resistance training and endurance training) on sleep parameters in athletes who might not be trained in both modalities, has not been addressed before.

Hyrox is a form of exercise training which has become a popular type of high-intensity functional training, consisting of blend elements of various training modalities including high-intensity interval training, strength training, running, and other endurance modalities (Davids 2025). Indeed, these “hybrid” athletes typically alternate between strength-focused and endurance-focused training sessions, characterized by completing physiological components representative of different sport training modes like maximal oxygen consumption, lactate threshold, maximal anaerobic power, as well as isometric and isokinetic strength and muscle power (Adami et al. 2021).

To the best of the authors’ knowledge, no study has investigated sleep characteristics in hybrid athletes, or focused on possible differences in sleep function after different training modes. It is hypothesized that following primarily endurance-training exercise bouts, sleep parameters will be impaired compared to resistance-training bout days. Secondly, training modes will demonstrate differential sympathetic activity, as reflected in the time and duration of cardiac arousals measured per bout. Therefore, this study aimed to objectively assess sleep quality and quantity in a sample of Hyrox athletes using an ecologically valid approach, to compare sleep characteristics following strength-based or endurance-based training bouts.

## Methods

### Design of the study and population

An ecologically valid, cohort, repeated-measures study was designed to investigate sleep characteristics in Hyrox athletes using a validated, wearable medical device for determining objective sleep measurements. The Institutional Review Board of the University of Trieste (122/230522) approved this study, which was conducted in accordance with the principles outlined in the Declaration of Helsinki. Participants completed written, informed consent. Before participating in the study, athletes were instructed not to modify their diet, training, or daily routine before or during the assessment period, which consisted of two weeks’ total duration.

The participants were recruited between mid-August and October 2025 from amongst a pool of experienced athletes training in the same “Hyrox” club, who met the following inclusion criteria: aged between 18 and 40 years, training for Hyrox competitions for at least one year, and performing at least one training per week dedicated to each exercise mode (i.e., at least one strength training/wk and one endurance trainings/wk). In alignment with previous protocols (Perrier et al. 2024), participants were excluded if they reported any current or recent injury within the last 14 days; these could include a major injury within the last month. Contraindications to study participation included: if athletes reported taking any drug or supplement affecting their sleep or circadian system (e.g., melatonin), cardiovascular medication, current dependence on alcohol, opiates, benzodiazepines, or if they drank more than 28 units of alcohol per week, smoked more than five cigarettes per day, or consumed more than 150 mg of caffeine per day. Finally, shift workers or those reporting a mean bedtime not falling between 09:30 PM and 12:30 AM were also excluded. To more accurately characterise the athlete sample in terms of their natural sleep habits, and to increase sample homogeneity, participants completed the Italian version of the Morningness-Eveningness Questionnaire (Cavallera and Boari 2015). Athletes were excluded if they were categorized as a “definite evening” type, since this classification may have affected their sleep quality measures (Lim et al. 2021), independent of training mode.

Training bouts were performed on the same day of the week, at the same time of day, which took place at some point between 09:00 AM and 07:00 PM, in accordance with the athlete’s regular training schedule. Training mode was completed in a counterbalanced order in order to reduce the risk of bias depending on intra-week based commitments. The training duration was the same cumulative exercise time for the ‘strength’ and ‘endurance’ focused training bouts. The athletes’ self-reported training duration ranged from 60 to 90 min per session (repeated identically, within-participant). An endurance training bout typically consisted of cycling or running at an intensity between 60 and 80% of the athlete’s maximum heart rate, whereas resistance training bouts typically consisted of completing fundamental strength training movements (e.g., squat, deadlift, etc.) between 60 and 80% of 1 repetition maximum, according to the athlete’s personal periodization routine. Since an ecologically valid approach was implemented in this investigation, a strict “time to bed” was not imposed, as long as the athletes were in bed before the 12:30 AM cut-off criteria time. Individual training routines, training duration, perceived exertion, and reported bed times are detailed in **Supplementary Table 1**.

### Sleep assessment and outcomes

Sleep quality and characteristics were assessed for the nights following 2 resistance and 2 endurance trainings (with 36–48 h of rest between them), performed within a two-week timespan, according to the design described above. A single-item sleep quality scale (SQS), based on a discretizing visual analog scale, was used to evaluate subjective sleep quality, which asked the participants to rate (within 2 h from waking up) their perceived sleep quality of the previous night. Data were based on a score ranging from 0 to 10, and categorized as follows: 0 = terrible, 1‒3 = poor, 4‒6 = fair, 7‒9 = good, and 10 = excellent using methods described elsewhere (Snyder et al. 2018). To objectively assess sleep parameters, participants used the Somno-Art system (PPRS, France), an ambulatory medical device consisting of an electronic armband that collects continuous physiological data, including heart rate, integrated with motor activity. Heart rate and derived heart rate variability have been suggested to be sleep-stage dependent, whereas actimetry (i.e. wrist movements) have been used as a proxy to assess total sleep time (Muzet et al. 2016). Collected data were analyzed by a dedicated software (Somno-Art Software v.2.8.0 [3.2.1]) which has been validated against gold-standard clinical polysomnography in both healthy and disturbed sleep populations (Thiesse et al. 2019, 2022a, b, 2023). The Somno-Art system was considered to be the best equipment available for use in this study because of its good (0.75 < ICC < 0.90) or excellent (ICC > 0.90) ICC scores it achieves for all sleep parameters, based on previous research which compares the device to clinical gold standard equipment. Importantly, N3 sleep is characterized as achieving only moderate acceptability, which should be noted when interpreting the results (0.50 < ICC < 0.75) (Thiesse et al., 2022). According to the manufacturer’s instructions, participants were instructed how to wear the armband device correctly before going to bed, and to effectively calibrate the device before going to bed (Lights Off). The recording was stopped when they woke up in the morning (Lights On). The extracted sleep parameters included: (i) sleep allocation time (min): duration from “Lights Off” to “Lights On”, (ii) total sleep time (TST, min): total duration of non-W (wake) stages, (iii) sleep latency (min): time from “Lights Off” to any sleep stage, (iv) wake after sleep onset (WASO, min): duration of W stages after sleep onset, (v) sleep efficiency (%): TST over sleep allocated time, (vi) latency R (min): time from sleep onset to first REM, (vii) N1 + N2 (min and %): total duration of non-REM stage 1 (N1) and non-REM stage 2 (N2), (viii) N3 (min and %): total duration of non-REM stage 3, and (ix) R (min and %): total duration of REM sleep. Additionally, the number and duration of cardiac arousals (CA, i.e., sudden increase in heart rate followed by a return to initial values) were also evaluated, in alignment with procedures described in detail elsewhere (Muzet et al. 2016). Cardiac arousals were recorded before and after sleep onset (SO). Since intraclass correlation analyses between the two nights of a given training mode revealed appropriate agreement (ICC ranges between 0.503 and 0.840), data obtained from the two individual training nights were averaged (within-mode) for statistical comparison between groups.

### Statistical analyses

To determine an appropriate sample size and achieved power, G*Power software was used. Expected values were based on data reported from similar previous work (Perrier et al. 2024), which employed 0.56 for the effect size (ES), an α of 0.05 and power of 0.8. From these assumptions, the minimum sample size needed to adequately power the current study was seven participants. This study is therefore adequately powered with eight participants completing all trials in a repeated-measures fashion. To confirm these statistical assumptions, a post-hoc analysis was performed which considered the significant outcomes with the smallest effect size (1.17), resulting in an achieved power of 0.81. Statistical analyses were performed using SPSS v.23 (IBM inc., USA). The Shapiro-Wilk test for normality of distribution was performed. Descriptive statistics were used to characterize the sample and summarize the results into medians and quartile ranges (1st quartile ‒ 3rd quartile) for continuous variables, and frequencies (percentages) for categorical variables. Paired-samples t tests were used to compare differences in mean sleep parameters between trials (resistance vs. endurance training mode), and Cohen’s *d* was reported as a measure of the effect size (ES), interpreted as follows: d = 0.2 small, 0.5 medium, 0.8 large (Cohen 1988). Bivariate correlation with Pearson’s coefficient (r) was conducted between sleep parameters of interest and cardiac arousals, both before and after sleep onset. Significance for statistical effects was set at *p* < 0.05.

## Results

Eight male hybrid athletes (aged between 23 and 32 years, median 28 years) volunteered for participation in the study and completed all trials, without any ‘dropouts’. Participants’ body mass was 75.8 kg (range: 60‒80), body height 1.80 m (range: 1.69‒1.85) and body mass index 23.3 kg/m^2^ (range: 20.2‒24.6). The athletes reported training histories specific to Hyrox competitions of between 1- and 6-years’ duration. According to their Morningness-Eveningness Questionnaire, five participants were categorized as ‘moderately evening’ type (score 31–41), 2 as ‘neither’ type (score 42–58) and one participant as ‘moderately morning’ type (score 59–69).

### Sleep outcomes

The median time between the end of training and “Lights Off” period was ~ 400 min (min/max range: 210‒555) and 425 min (min/max range: 295‒480) after resistance training and endurance training, respectively. On average across all trials, Hyrox athletes self-reported relatively “good” perceived sleep quality (80% of the nights scored as “good” and 20% of the nights scored as “fair”), with a median sleep allocation time (determined via SomnoArt) of 454 min (range: 439‒477), TST of 394 min (range: 381‒407), sleep latency of 15 min (range: 10‒31), WASO of 39 min (range: 30‒49), sleep efficiency of 86% (range: 85‒87) and latency R of 75 min (range: 68‒86). Considering sleep architecture, wakefulness (W) was 62 min (range: 58‒69), light N1 + N2 sleep was 62% (range: 59‒63), deep N3 sleep was 18% (range: 17‒21), and Rapid-Eye Movement R sleep was 20% (range: 19‒21). Notably, before sleep onset, the number of cardiac arousals (CA) was 7 (range: 5‒13), lasting ~ 2 min (range: 2‒4), whereas after sleep onset, the number of CA was dramatically higher, at 94 (range: 79‒103), and lasting longer, to ~ 31 min (range: 29‒37).

**Fig. 1:**
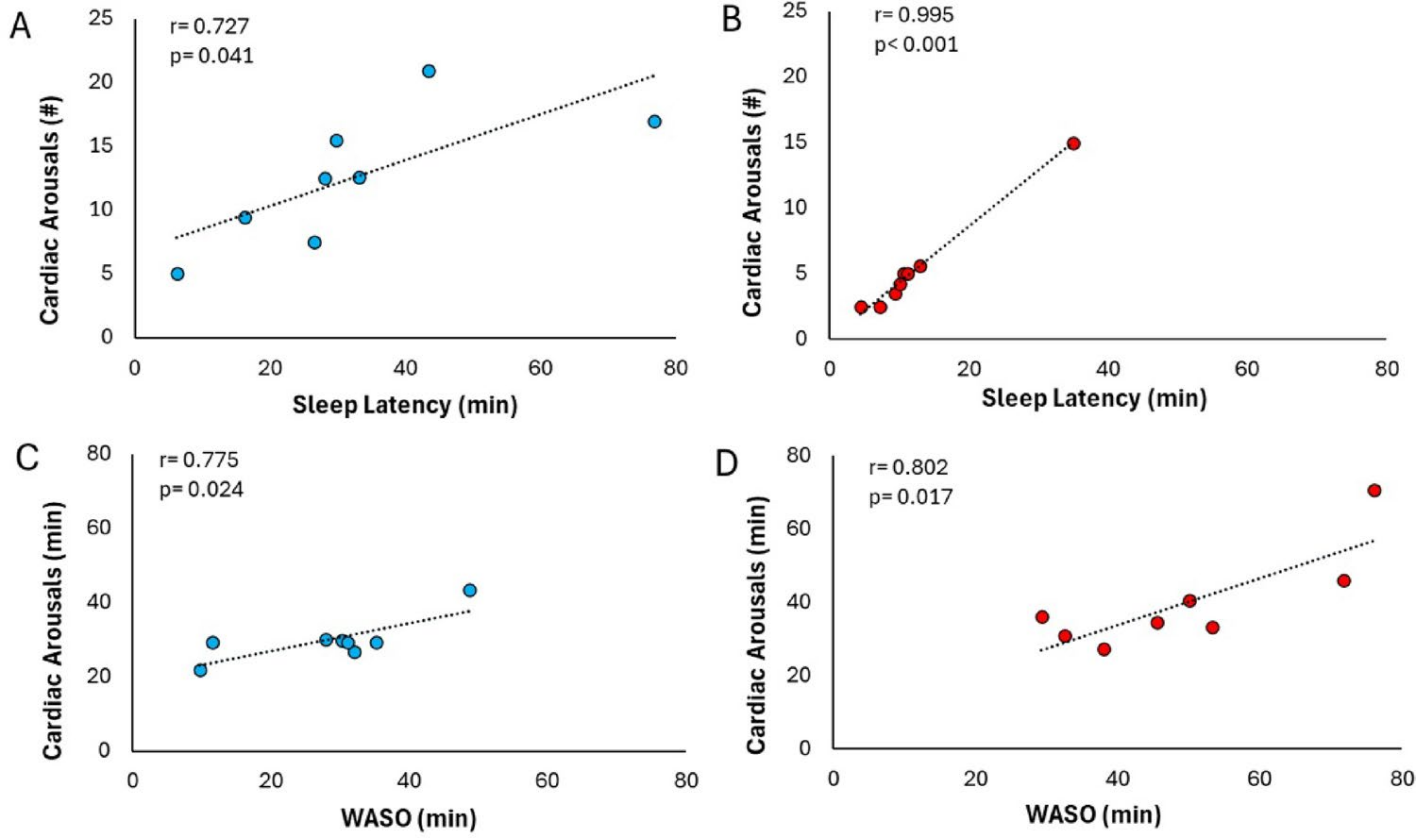
Bivariate correlation analyses between **A** sleep latency after resistance training and cardiac arousals (number, counts, blue circles) before sleep onset, **B** sleep latency after endurance training and cardiac arousals (number, counts, red circles) before sleep onset, **C** between wake after sleep onset (WASO) after resistance training and cardiac arousals (duration, min, blue circles) after sleep onset, and **D** between WASO after endurance training and cardiac arousals (duration, min, red circles) after sleep onset. Pearson’s correlation coefficient r and significance are labelled directly on the figures

Between-mode exercise comparisons of all sleep parameters (i.e. resistance training versus endurance training) is reported in Table 1. Correlation analysis determined there were significant associations in sleep latency scores and the number of CA before SO following resistance training (*r* = 0.727, *p* = 0.041) (Fig. 1**A**) as well as after endurance training (*r* = 0.995, *p* < 0.001) (Fig. 1**B**). There were also significant associations present between WASO and the duration of CA after SO following resistance training (*r* = 0.775, *p* = 0.024) (Fig. 1**C**) and after endurance training (*r* = 0.802, *p* = 0.017) (Fig. 1**D**).

## Discussion

Sleep is a key behavioural health factor which affects athletic performance, including ability to complete high training loads with appropriate intensity, concentration and vigor; nevertheless, few information is known about the effects of different training modalities on sleep outcomes in competing athletes. To the best of the authors’ knowledge, this is the first study to investigate objectively-measured sleep characteristics in hybrid athletes, including a comparison between two training modes (i.e., resistance training and endurance training). All Hyrox participants presented with generally ‘good’ sleep outcomes overall, according to both subjective and objective parameters. There were slight differences between training modes in terms of sleep outcomes, which depended on the training mode, affecting one’s ability to fall asleep and cardiac characteristics also differed between exercise modes.

In the present study, hybrid athletes were characterized by performing specific, repeatable exercise training bouts across the study period (see: **Supplemental Table 1** for training details). Briefly, although this study applied an ecologically-valid design (i.e. did not impose specific training loads per se), each athlete was interviewed to determine what was their training routine, and whether stark differences were present between the participants; all athletes reported similar intensities and duration of trainings across the week in terms of %HR max (for the endurance training) and %1RM (for the resistance training). Of relevance, the endurance and strength training loads were identical within the two recording days.

According to a recent study conducted with 175 young adult élite athletes from 12 different sports (Sargent et al. 2021), researchers reported actigraphy-based habitual sleep duration was 6.7 ± 0.8 h, which was shorter in athletes from individual sports compared to those participating in team sports. Athletes self-reported sleep satisfaction scores of 6.8 ± 1.6/10.0. In another cross-sectional study on 313 athletes (aged between 18 and 60 years), and recruited from a variety of sport disciplines (Randell et al. 2021), self-reported sleep duration was 7.6 ± 1.0 h, with lower sleep durations found in runners compared to team sports athletes. For this sample, sleep onset latency was reported as ~ 22 min. A systematic review on 1830 athletes reported average sleep duration of 7.7 ± 1.1 h and 86.3 ± 6.8% sleep efficiency (Vlahoyiannis et al. 2021), whereas portable polysomnography was recently used to assess sleep parameters in 13 élite youth rowers (Hof zum Berge et al. 2021a), finding a total sleep time ~ 6.7 h, sleep onset latency ~ 18 min, WASO ~ 42 min and sleep efficiency ~ 82%.

Athletes reported a total sleep time of ~ 6.6 h, which is in line with the previous literature, in particular with the previous study in rowers (Hof zum Berge et al. 2021a). Other sleep parameters assessed sleep onset latency (~ 15 min), WASO (~ 39 min), and sleep efficiency (~ 86%), which were similar to those previously reported, although differences were found according to the type of training; resistance training was characterized by higher sleep onset latency, but lower WASO compared to endurance training. To date, the only study directly comparing the effects of strength and endurance acute exercise on sleep parameters did not report any significant differences between the two types of training; however, participants were healthy trained males who had not actively trained in hybrid disciplines. The training sessions also started at 10:00 (Roveda et al. 2011), potentially only minimally affecting subsequent sleep. The possible effect of resistance training on sleep latency may be supported by previous data suggesting that, according to the type of sport, sleep onset latency is longer in power/explosiveness disciplines compared to other sports (Vlahoyiannis et al. 2021). Moreover, impaired WASO and sleep onset latency have been found to be correlated with daily training load (de Blasiis et al. 2021), although the role of exercise-induced delayed onset muscle soreness is still debated (Hausswirth et al. 2014; de Blasiis et al. 2021).

Considering sleep architecture, our study found that deep sleep stage (N3) accounted for 18% (range: 17‒21) of sleep time, and a proportion of REM sleep of 20% (range: 19‒21); these values are consistent with a previous study on 20 élite athletes from five different sports disciplines (Hof zum Berge et al. 2021b), which found sleep architecture proportions of 5.73 ± 3.41% N1, 36.78 ± 7.67% N2, 26.4 ± 6.13% N3, and 8.02 ± 5.18% of RM sleep. Compared tonon-athletic adult populations who typically spending more time in non-REM sleep and less time in REM sleep, hybrid athletes spent ~ 5% less time in REM, but ~ 7% longer time in N1 + N2. No significant differences were found according to training mode, although in the literature the type of sport has been suggested to potentially affect sleep architecture, since anaerobic sports is characterized by slightly less REM sleep, whereas N3 sleep is reportedly longer in athletes in mixed sports than in athletes in aerobic and power sports (Vlahoyiannis et al. 2021). Finally, seminal works in sport literature have found that after endurance races, REM proportions can be reduced and N3 stage sleep durations may increase, thereby suggesting REM sleep as a sensitive indicator of exercise-induced stress (Shapiro et al. 1981; Driver et al. 1994), while N3 sleep increases in response to higher physiological restorative demand in athletes (Sekiguchi et al. 2019).

Cardiac autonomic activity is potentially affected by the type of exercise training. In the present study, cardiac arousals were more frequent and longer before sleep onset after completing a bout of resistance training exercise, whereas more frequent and longer cardiac arousals were found to occur after sleep onset when endurance training was performed. These findings seem to provide an autonomic rationale of differential effects of the training modality on sleep latency (i.e., longer after resistance training) and WASO (longer after endurance training), although the causative nature of this relationship cannot be confirmed by correlation analysis alone. Despite its possible relation to sleep onset and maintenance (Nano et al. 2020), the role of cardiac autonomic response during sleep has been scarcely investigated after performing exercise. It has been found that with late exercise timing and high exercise strain, there are potential associations with higher nocturnal resting heart rate and lower nocturnal heart rate variability (i.e., higher sympathetic drive) (Leota et al. 2025). These responses may be exacerbated, or more evident after vigorous late-night exercise during the first sleeping hours (Myllymäki et al. 2011). As such, this study confirms previous works which have suggested an association between cardiac autonomic activity and sleep quality, and may be of interest to the athletic population due to this modulation based on exercise-mode. Taken together, the findings communicated in this work suggest that hybrid athletes present with sleep characteristics that are in line with those found in other “classical” sport disciplines, although the type of training (resistance training vs. endurance training) appears to influence sleep quality, with a possible effect on cardiac autonomic activity observed both before and after sleep onset.

This study encompassed an ecologically valid design, which included athletes who had similar training characteristics, and without imposing specific regimens that might have affected their normal training routine or normal sleep function. The study therefore employed a cross-over design in random training order to minimize any potential bias depending on the days of the week, or order effect on sleep function. However, due to the participants’ tight training schedule, it was not possible to compare sleep outcomes after training days to sleep outcomes after full rest days, without influencing the athlete’s training schedule and periodization plans directly. Since this study has attempted to examine differences based on exercise mode, we did require somewhat comparable ‘workloads’ or ‘work completed’ bouts, which were generally compatible, although it remains that direct comparison between exercise modes could not take into account all real-world training complexities (e.g. nutritional loading, hydration status, energetics). Every effort was made to ensure a fair comparison was possible, and this effort was done by minimizing external factors researchers could control, e.g. requesting athletes to complete comparable overall bout durations, completing two-days with identical training and nutrition logs for a given mode, and ensuring all exercise was completed several hours before bedtime. Further, to reduce the influence of different chronotypes on sleep quality, “definitely evening” types were not included in this study, which might limit the generalizability of the results to athletes with such a chronotype. In the current study, only male participants volunteered for the study and were included in the final data analysis, therefore limiting the generalizability of these findings to female athletes. The researchers recognize far more studies are needed to clearly identify the influence of menstrual cycle and female-specific physiology on sleep and performance outcomes in female athletes (Hrozanova et al. 2021; Kullik et al. 2025). The use of a validated wearable medical device for objective sleep measurements allowed us to collect several data about sleep quality, quantity and architecture, with a minimally invasive protocol. Finally, the focus on hybrid athletes not only provided novel data in an understudied trained population, but also provided an insight into the influence of the type of training on sleep in athletes used to both training modalities. The findings presented herein suggest i) typical training routines in hybrid athletes do not impair sleep quality or quantity per se, at least when trainings are completed more than 3–4 hours before going to bed, however ii) cardiac arousals ( a sign of sympathetic activation) might affect sleep latency and “wake after sleep onset” parameters, so promoting relaxation practices, and good sleep hygiene techniques, are critical for athletes to encourage or improve sleep quality when training vigorously.

## Conclusion

Hyrox athletes are characterized by high training loads and different training modes, mainly resistance training and endurance training, which differentially affected the athletes’ sleep quality and quantity. From this preliminary study, hybrid athletes presented sleep characteristics in line with those found in other sport populations, and in general, only slightly poorer sleep parameters compared to non-athletes. Resistance training increased sleep onset latency while endurance training increased the time wake after sleep onset compared to the other exercise mode, which was reflected in an increased cardiac autonomic activity before and after sleep onset, respectively.

**Table 1:** Median (1st quartile**‒**3rd quartile) **s**leep characteristics of the included sample (*n* = 8) after resistance training and endurance training

	Resistance training	Endurance training	Significance (ES)
SQS Sleep allocation time, min TST, min Sleep latency, min WASO, min Sleep efficiency, % Latency R, min W duration, min N1 + N2, % N3, % R, % Number of CA before SO Duration of CA before SO, min Number of CA after SO Duration of CA after SO, min	8 (7‒8) 449 (439‒466) 388 (374‒399) 29 (24‒36) 31 (24‒33) 87 (86‒88) 76 (73‒79) 61 (58‒67) 61 (59‒62) 19 (18‒21) 21 (20‒21) 13 (9‒16) 4 (4‒5) 81 (67‒93) 30 (29‒30)	8 (7‒8) 458 (437‒486) 399 (388‒415) 10 (9‒12) 48 (37‒58) 86 (85‒87) 71 (66‒94) 64 (58‒71) 63 (61‒63) 18 (18‒20) 19 (18‒20) 5 (3‒5) 2 (2‒2) 99 (95‒106) 36 (33‒42)	0.451 (0.28) 0.404 (0.32) 0.508 (0.25) **0.003 (1.55)** **0.008 (1.30)** 0.669 (0.16) 0.452 (0.28) 0.774 (0.10) 0.310 (0.39) 0.690 (0.15) 0.120 (0.64) **0.006 (1.38)** **0.005 (1.43)** **0.010 (1.26)** **0.013 (1.17)**

Single-item sleep quality scale (SQS), total sleep time (TST), wake after sleep onset (WASO), rapid eye movement sleep (R), non-REM sleep stage 1 (N1), non-REM sleep stage 2 (N2), non-REM sleep stage 3 (N3), cardiac arousal (CA), sleep onset (SO). Bold values for *p* < 0.05 at the paired samples t-test. ES: effect size, Cohen’s *d*

## Supplementary Information

Below is the link to the electronic supplementary material.

Supplementary Material 1

## Data Availability

anonymized data can be requested upon reasonable request to the corresponding author.

## Abbreviations

CA: cardiac arousals
HIE: high intensity exercise
R / REM: rapid eye movement stage
SO: sleep onset
SOL: sleep onset laten
TST: total sleep time
W: wake stage
WASO: wake after sleep onset
