# Supplementary material for: Strengthening recovery, enduring sleep. An ecologically valid assessment of sleep quantity and quality in hybrid athletes: does training mode matter?
Source: Eur J Appl Physiol. 2026 Feb 28;126(6):3473–80. doi: 10.1007/s00421-026-06187-9 (PMC13287113; doi:10.1007/s00421-026-06187-9)
Supplement: Supplementary file 1 — Supplementary Material 1 [file 421_2026_6187_MOESM1_ESM.docx]

| **Participant** | **Resistance training** | **Endurance training** |
| --- | --- | --- |
| 1 | End of training: 12:00, Bed time: 23:00  *Training routine* (**RPE: 7, duration: 80 min**): fundamentals 4 sets x 2 reps at 85% 1RM, other exercises 3 x 10-12 reps at 50% 1RM | End of training: 10:00, Bed time: 23:30  *Training routine* (**RPE: 7, duration: 70 min**): sprint running, interval training 6 x 800 m at 75-85% HRmax, interval training 4 x 1500 m at 60-70% HRmax |
| 2 | End of training: 18:00, Bed time: 23:30  *Training routine* (**RPE: 7, duration: 75 min**): fundamentals 4 x 1 rep at 90% 1RM, accessory lifts 3 x 6-8 reps at 60-75% 1RM | End of training: 15:00, Bed time: 22:30  *Training routine* (**RPE: 7, duration: 75 min**): cycling at 65-75% HRmax |
| 3 | End of training: 17:30, Bed time: 23:00  *Training routine* (**RPE: 5, duration: 60 min**): power exercises (e.g., squat jumps) 3 x 5 reps at 10-20% 1RM, other exercises 4 x 8-10 reps at 50% 1RM | End of training: 17:30, Bed time: 22:45  *Training routine* (**RPE: 6, duration: 70 min**): running 3 x 15 min in zone 3, 8 x 2 min at anaerobic threshold |
| 4 | End of training: 18:00, Bed time: 22:30  *Training routine* (**RPE: 6, duration: 80 min**): power exercises (e.g., squat jumps) 3 x 5 reps at 10-20% 1RM, other exercises 4 x 8-10 reps at 60% 1RM | End of training: 12:00, Bed time: 23:00  *Training routine* (**RPE: 7, duration: 70 min**): running 2 x 20 min zone 2, 2 x 10 min zone 3 |
| 5 | End of training: 18:30, Bed time: 22:00  *Training routine* (**RPE: 6, duration: 75 min**): fundamentals for strength 3 x 4-6 reps to failure and “metabolic circuits” | End of training: 14:00, Bed time: 22:30  *Training routine* (**RPE: 6, duration: 60 min**): running on perceived exertion |
| 6 | End of training: 18:00, Bed time: 23:00  *Training routine* (**RPE: 6, duration: 80 min**): Olympic weightlifting exercises at 50% 1RM, 2-3 x 10 reps, deadlift and bench press 2 x 4 reps at 80% 1RM | End of training: 12:00, Bed time: 22:45  *Training routine* (**RPE: 6, duration: 70 min**): running 30 min zone 2, interval training 4 x 800 m at 75% HRmax |
| 7 | End of training: 14:00, Bed time: 22:30  *Training routine* (**RPE: 6, duration: 75 min**): fundamentals 4 x 2 rep at 90% 1RM, accessory lifts 4 x 8 reps at 65-75% 1RM | End of training: 18:00, Bed time: 22:45  *Training routine* (**RPE: 7, duration: 60 min**): running, 8 km zone 3, 2 km zone 2 |
| 8 | End of training: 10:00, Bed time: 22:00  *Training routine* (**RPE: 7, duration: 60 min**): weighted calisthenics workout | End of training: 17:30, Bed time: 22:15  *Training routine* (**RPE: 6, duration: 60 min**): running, 5 km zone 3, interval training 4 x 400 m at 85% HRmax |

**Supplementary Table 1.** Training routines of the participants during the study. Each training period lasted between 60- and 90-min duration.

**Notes:** RPE: rate of perceived exertion (1-10); 1RM: 1-Rep Max is the maximum amount of weight a person can lift for a single, proper-form repetition of an exercise; HRmax: maximum heart rate (estimated)
